# Society Meetings

**Published:** 1901-10

**Authors:** 


### SOCIETY MEETINGS.

THE American Association of Obstetricians and Gynecologists held its fourteenth annual meeting at Cleveland, O., September 17, 18 and 19, 1901. The president, Dr. William E. B. Davis, of Birmingham, conducted the meeting most efficiently and it easily takes rank as one of the best in the history of the association. The attendance both of Fellows and visitors was unusually large and the debates were spirited, instructive and entertaining.

The arrangements for the meeting were most admirably made and carried out, reflecting great credit upon the local committee in charge, which consisted of Dr. M. Rosenwasser and Dr. W. H. Humiston. A proposition made last year to change the name of the association to The American Society of Abdominal Surgeons was lost, it having received but two votes,—those of the mover and the seconder. A committee of three, consisting of Drs. Davis, Carstens and Dunning, was appointed to confer with the Southern Surgical and Gynecological Association on the subject of a union of the two bodies,—the report to be made next year.

The annual dinner served at The Hollenden was a most interesting occasion. The tables were handsomely laid and the room was appropriately decorated. The occasion was made largely a memorial to President McKinley, the speakers having been chosen with that object in view, and was presided over by Dr. W. H. Humiston. The address of Hon. J. H. Hoyt, of Cleveland, to the subject, "Our Martyred President," was an eloquent, graceful and touching tribute to the memory of the President who was his neighbor and personal friend. Among the other sentiments of similar tone were "The Late President and the New South," responded to by Dr. L. S. McMurtry, of Louisville; and "Modern Surgery and the President's Case," responded to by Dr. W. G. Macdonald, of Albany. Both of these speakers did themselves and the occasion great credit in the substance, manner and method of their addresses: indeed, all the speeches were models of good form.

Officers were elected for the ensuing year as follows: president, Edwin Ricketts, Cincinnati; vice-presidents, Charles G. Cumston, Boston, and Miles F. Porter, Fort Wayne; secretary, William Warren Potter, Buffalo; treasurer, X. O. Werder, Pittsburg; executive councillors, to succeed themselves, L. H. Dunning, Indianapolis, and Walter B. Chase, New York; to fill a vacancy, William H. Humiston, Cleveland.

The next meeting was appointed to be held at Washington, beginning the third Tuesday of September, 1902. The association adjourned without day at half-past one o'clock Thursday, out of respect to the memory of the late President, whose funeral services at Canton were to commence at two o'clock.

---

The Buffalo Academy of Medicine held meetings during the month of September, 1901, as follows:

*Section on Medicine.*—Tuesday evening, September 3. Program: Intestinal dystrypsia, J. C. Hemmeter, Baltimore, Md. Treatment of pneumonia, DeLancey Rochester.

*Section on Surgery.*—Tuesday evening, September 10. Program: Some points on resection of the hip and knee joints, Herman Mynter. Meeting postponed on account of the essayist being in attendance upon the President.

*Section on Obstetrics.*—Tuesday evening, September 24. Program: Some experiences in a series of obstetrical cases,

Eli H. Long. Diagnosis in vertex presentations, P. W. Van Peyma.

*Section on Ophthalmology, Otology, Laryngology, and Rhinology.*—Monday evening, September 16. Program: Methyl alcohol, should it be placed on the list of poisons? Swan M. Burnett, Washington, D.C.

*Section on Pathology.*—Tuesday evening, September 17. Program: Some demonstrations, with projecting microscope, of embryological specimens, including some abnormalities, Prof. Simon Harry Gage, Cornell University, Ithaca,

---

THE 73d annual meeting of the German Nationalists and Medical Congress was held at Hamburg, Germany, September 22 to 28, 1901. An elaborate program was prepared and great preparation made to hold one of the greatest meetings in the history of the society. This corresponds to the American Medical Association and Association for the Advancement of Science combined, and hence some idea of its scope and strength can be gained by the comparison.

---

THE Medical Society of the State of New York will hold a semi-annual meeting in Hosack Hall, New York Academy of Medicine, No. 17 West 43d. Street, New York City, on October 15 and 16, 1091. An excellent program has been prepared and a large attendance is anticipated.

